# Supplementary material for: Are carbon emissions trading and green financial instruments synergistic? -Comprehensive quantitative research based on content analysis
Source: PLoS One. 2024 Mar 7;19(3):e0298601. doi: 10.1371/journal.pone.0298601 (PMC10919601; doi:10.1371/journal.pone.0298601)
Supplement: S1 Data — (ZIP) [file pone.0298601.s001.zip › Data/地级市Z_F工作报告.zip!/╔╜╢1⁄2/╬1⁄2╖╗╩╨/└┤╫╘╣1⁄2╓┌║┼í╛┬φ┐╦╩2╛▌═°í┐_2016.docx]

各位代表:

现在,我代表市政府向大会报告工作,请予审议,并请市政协委员和其他列席会议的同志提出意见。

一、2015年经济社会发展计划和“十二五”规划完成情况

2015年是“十二五”规划的收官之年。面对宏观经济下行和错综复杂的国内外形势,在市委的坚强领导下,市政府与全市人民一道,坚持稳中求进的工作总基调,统筹推进稳增长、调结构、促改革、惠民生、防风险各项工作,圆满完成了市十六届人大四次会议确定的目标任务。全市地区生产总值增长8.3%,一般公共预算收入增长12.6%,社会消费品零售总额增长10.6%,固定资产投资增长13.9%,进出口总额增长6.5%,城镇和农村居民人均可支配收入分别增长8.3%、9.2%,实现了“十二五”圆满收官,全市经济社会发展实现了新的历史跨越。

综合经济实力迈上新台阶。“十二五”期间,全市地区生产总值连续跨越4000亿元、5000亿元两个台阶,达到5170.5亿元,年均增长9.9%;人均地区生产总值在全省提高两个位次。一般公共预算收入连续跨越300亿元、400亿元两个台阶,达到484.5亿元,年均增长19.1%。固定资产投资、社会消费品零售总额分别达到4516.7亿元、2277.5亿元,分别增加了0.96倍和0.98倍。各类市场主体由30.9万户增加到64.8万户,增加了1.1倍,总量居全省第2位。

人民生活水平得到新提升。城镇和农村居民人均可支配收入分别达到31060元、14890元,分别比2010年增长了58%和68%。累计实施民生实事103件。民生总支出1873.2亿元,占财政支出的比重由73.4%提高到79%。减少贫困人口30.5万人。城乡居民基本养老和基本医疗保险实现并轨。居民供水、医疗救助实现城乡一体化。新增城镇就业及农村劳动力转移就业117.7万人。每千名老人养老床位由20张增加到31张。全市中小学标准化率由11%提高到97%,国家义务教育发展基本均衡县实现全覆盖。四级公共文化服务网络基本建成。城、乡人均住房面积分别达到41.5㎡和41㎡。城镇每百户拥有生活用汽车由18.8辆提高到66.6辆,农村由11辆提高到37辆。互联网普及率达到71%。人均预期寿命由77.95岁提高到78.5岁,高于全国2.7岁。

经济结构调整取得新进展。三次产业比例由10.7:55.6:33.7调整到8.8:48.2:43。高新技术、新兴产业产值占比分别达到31.9% 和13.3%。新增各类农业规模经营主体22116家、现代农业示范园区912处,63.9%的土地实现规模化经营。规模以上工业主营业务收入和利税分别达到1.3万亿元、1115亿元。新增各类金融机构162家、境内外上市公司7家,引进各类总部及分支机构1028家。5A级物流企业、5A级旅游景区、国际品牌五星级酒店、国际产品交易平台实现了零的突破。

发展动力转换取得新成效。全社会研发投入占GDP比重达到2.5%。境外研发机构发展到25家,引进各类院所及分支机构92家。新建成1个企业国家重点实验室、2个国家级工程技术研究中心、4个国家产业技术创新战略联盟。福田汽车研究院、潍坊创业大学、中美技术转移中心、潍坊(美国)硅谷高科技孵化器、中以跨境多币种科技创新基金、3D打印暨先进制造中心、蓝色智谷等一批高端创新创业平台建成投用。新增各类孵化器163.8万㎡。获授权国内专利 39985件,其中发明专利3343件,申请国际专利371件。发放小额担保贴息贷款46.6亿元,扶持14.4万人自主创业就业。驰名商标达到127 件,增长154%。列《福布斯》2015中国最佳商业城市百强第32位。

体制机制创新实现新突破。省部共建国家职业教育创新发展试验区取得重大突破。市区管理体制进一步理顺。县级公立医院综合改革全面启动。现代农业综合改革试点深入推进,农民承包地、宅基地和农村集体建设用地确权登记颁证工作率先完成,农村产权交易中心建设、农村集体产权制度改革、新型农村合作金融试点、供销社综合改革试点扎实推进。率先推行财政产业专项资金基金化管理改革,市级政府引导基金规模达到24亿元,设立产业基金29支,撬动金融和社会资本 315亿元。大力推行“PPP”融资机制,成功运作项目19个。市县两级政府均设立融资担保基金,总规模达到38.96亿元。财政透明度位居全国前列。先后探索实行产业竞争性用地、工业供地“先租后让”“长期租赁”“弹性年期出让”、房屋征收“裁执分离”、按行业环保先进标准管理重大项目、非制造业建设工程项目审批告知承诺制等一系列重要改革措施。建设工程项目审批由86天缩减为35天。2015年民营经济发展环境综合评价列全省第1位。

开放发展开创新局面。进出口总额从全省第5位跃居第3位,达到189亿美元,增加了0.6倍。五年累计实际到账外资42亿美元。建立海外工业园、能源资源供应基地10个,境外营销机构188家,新引进世界500强企业30家。潍坊出口加工区升级为全国第14个综合保税区,峡山区获批建设山东第一个生态经济区。东亚畜牧产品交易所、进口澳大利亚活体肉牛指定口岸获批建设。跨境电子商务平台顺利通过验收。成功举办风筝会、鲁台会、中国画节和首届中日韩产业博览会。

区域统筹发展趟出新路子。突破滨海、提升市区、开发两河扎实推进。涉海经济规模突破4000亿元。潍坊港吞吐量突破3000万吨。潍坊港国际集装箱航线、鲁辽陆海货滚甩挂运输大通道开通运营,潍坊渔港开港运行。市区“两廊两核四区多园”产业格局加快形成,教育、医疗等“六大服务功能”快速提升,中小学建设改造、“四通一治”等“七项工程”扎实推进,共实施各类提升项目313个、总投资415.6亿元。成功创建为全国文明城市,实现了潍坊近十年的夙愿。 “两河”开发总体规划和6个专项规划编制完成。开工建设“十大工程”275个项目,总投资143亿元。流域生态治理、道路桥梁建设、南部山区保护开发取得阶段性成果。全市现代化综合交通、水利、能源保障体系加快形成。德大铁路及长深高速建成投运,济青高铁、潍日高速、黄大铁路、青银高速改造工程开工建设, 通过潍坊的京沪高铁东线列入省“十三五”规划,潍坊机场实现对外开放。国华寿光电厂一期建设完成。引黄入峡、引黄入白工程建成运营,成为我市特大干旱情况下用水安全的根本保障。全市常住人口城镇化率达到55.8%,提高了8.8个百分点。

生态文明建设实现新突破。绿色生产、绿色建筑、绿色交通加快推进。完成建筑节能改造566万㎡。市区清洁能源公交车达到935台。投入运营公共自行车 3.24万辆,年骑行量达到6032万人次,成为我市绿色文明的一道靓丽风景线。“三八六”环保行动圆满收官,三年共实施环保项目1668个,拆改燃煤锅炉538台,关停环保不达标企业和生产线829个(条),拒批缓批项目251个。全市生态环境质量有了根本改善,“蓝天白云、繁星闪烁”天数由2013年的150天增加到234天,跃升到全省第5位。PM2.5、SO2、NO2分别比2013年改善25.2%、42.8%和14.8%。

民主政治和社会治理再上新水平。坚持党的领导、人民当家作主、依法治国有机统一。认真执行市人大及其常委会决议决定并自觉接受其监督,把政治协商纳入决策程序,共办理人大代表议案建议和政协委员提案2307件。

《法治潍坊建设纲要》印发实施,法治体系更加完善。获得地方立法权。在全省率先发布政府部门权力清单、服务清单、责任清单,累计下放市级审批事项 189项。全面推行重大行政决策合法性合理性审查、听证、风险评估等制度和政府顾问制度。基层治理体系更加完善,平安潍坊建设成效显著。国家食品安全城市创建试点工作“三项评估”均列全国前茅。严格贯彻落实中央八项规定精神和省市相关规定,扎实开展党的群众路线教育实践活动和“三严三实”专题教育。党风廉政建设和反腐败工作深入推进,全市纪检监察机关共审查违纪案件8909件,党纪政纪处分8232人。

这些成绩是在宏观经济持续下行、我市遭遇有水文记载以来最严重旱情、铁腕治污、大力整治安全生产隐患的情况下取得的,体现了速度与质量、规模与结构效益、生产与生活生态、抓当前与利长远的有机统一。这是省委、省政府和市委坚强领导的结果,是市人大、市政协和社会各界同心同德、大力支持的结果,是全市广大干部群众、驻潍部队和武警官兵团结奋斗、开拓进取的结果,也是在外潍坊籍乡亲和海内外朋友关心支持的结果。在此,谨代表市政府表示衷心的感谢和崇高的敬意!360小说

我们也清醒地看到,我市经济社会发展还存在许多短板:人均发展水平还不够高,大而不强的问题仍然突出。社会治理能力和治理体系现代化建设任重道远。经济结构不优的问题仍然突出;发展还不够平衡、协调;基础设施还不完善;土地、水、环境等约束趋紧,旱情仍在持续,水资源浪费的现象还大量存在。部分群众生活仍然困难,仍有20.3万人生活在省定贫困线以下;部分领域潜在风险加大;医疗、养老、生态、城市交通等优质资源供给不足。政府机关及工作人员作风不严不实甚至腐败问题依然存在。对这些问题,我们要高度重视,认真解决。二、“十三五”经济社会发展的主要目标和重点任务

“十三五”是全面建成小康社会的决胜阶段,也是我市转型发展的关键时期。要全面落实市委十一届八次全体会议精神,协调推进“四个全面”战略布局,坚持发展第一要务,以创新、协调、绿色、开放、共享发展理念为引领,以提高发展质量和效益为中心,坚持稳中求进、以转促进、统筹发展,深入推进“四一三”战略重点和区域统筹发展布局,努力在全面建成小康社会进程中走在前列。努力实现以下目标:

——综合实力迈上新台阶。全市地区生产总值达到7500亿元,年均增长8%左右,人均地区生产总值达到1.3万美元以上,提前实现经济总量和城乡居民人均收入比2010年翻一番。产业迈向中高端水平,现代产业体系更加完善,产业竞争力明显增强。

——创新能力明显提升。构建起充满活力、富有效力的创新体制机制,全社会研发投入占比达到2.8%,科技创新的引领作用更加凸显,新技术、新产业、新业态蓬勃发展,创新型城市建设达到新水平。

——人民生活水平和质量明显提高。基本公共服务更加均等,社会保障能力明显增强,教育、健康水平和生活环境质量明显提升。改革发展成果更多更公平惠及全市人民。人均预期寿命提高到79.5岁以上。

——国民素质和社会文明程度全面提升。社会主义核心价值观更加深入人心,覆盖城乡的公共文化服务体系基本建成,市民思想道德素质、科学文化素质、健康素质、法治素养明显提升。文化产业成为支柱产业。

——发展的协调性全面增强。突破滨海、提升市区、开发两河取得明显成效,以人为核心的新型城镇化水平大幅提升。中心城市吸引力、辐射带动能力有新的跨越,滨海新区建成全国知名的现代化海洋经济新区。县城、小城镇、农村社区承载服务能力进一步增强。常住人口城镇化率达到65%左右。

——生态环境质量显著改善。大气、水、土壤环境质量持续改善,山水林田湖生态系统得到有效修复保护,城市PM2.5平均浓度下降到0.049mg/m3,“蓝天白云、繁星闪烁”天数稳定在252天以上。绿色生产方式、生活方式基本形成,生态美丽新潍坊形象进一步展现。

——体制机制更加健全。重点领域和关键环节改革取得决定性成果。开放型经济新体制更加完善,国际化水平大幅提升。社会治理体系和治理能力现代化取得重大进展,人民民主更加健全,法治政府基本建成。

各位代表,要实现“十三五”宏伟目标,必须坚持创新发展、协调发展、绿色发展、开放发展、共享发展。

坚持创新发展。把创新作为发展第一动力,作为结构性改革根本措施,在全社会大力弘扬创新精神,传播创新文化,树立“人人可创新、事事可创新、改进即创新”理念,营造全社会崇尚、尊重、支持、参与创新创造的浓厚氛围,形成大众创业、万众创新的生动局面,建设创新型城市。突出科技创新。加大政府支持力度, 落实企业主体地位和人才核心地位,围绕构建现代产业体系构建创新体系、人才体系。加快建设现代产业体系。大力发展品牌农业,引领农业由产量规模型向质量效益型、由生产型向一二三次产业融合发展的历史跨越。落实藏粮于地、藏粮于技战略,调整种养结构,改良土壤,突破种业,提升新型农业经营主体科学经营能力, 加快农业信息化、品牌化、国际化步伐。实行最严格的农产品质量管理,争创国家农产品质量安全市。实施工业转型升级五年计划,落实“中国制造2025”潍坊行动方案、“互联网+工业”行动方案、制造服务化五年行动方案,推动传统制造业向中高端迈进,新兴产业由小到大跨越。到“十三五”末,工业主营业务收入突破2万亿元,新兴产业及高新技术产业占比达到50%以上,工业单位能耗、水耗及排放明显下降,综合质量效益明显提升,打造电子信息、生物医药、生物基材料三个新的千亿级产业链,智能装备、新能源及电动汽车两个300亿级产业链。实施新一轮服务业跨越发展计划,集中突破总部经济、金融、物流、电子商务、服务外包等生产性服务业,加快发展健康、养老、文化、旅游、家政等生活性服务业,拓展国际服务贸易和农村服务业,服务业增加值达到3700亿元、占比提高到 50%以上。实施重大基础设施提升行动,五年完成基础设施投资9000亿元以上。潍坊港建成亿吨大港,新机场建成投用,济青高铁、潍莱高铁、疏港铁路、潍日高速、城海轻轨建成运营,京沪高铁东线加快推进。市区内“五横五纵”快速通道及与周边六市(县、区)快速连接通道基本建成,市区轨道交通1号线、2号线加快建设,全市现代化综合交通体系基本形成,把潍坊打造成为国内知名的现代化综合交通枢纽城市。能源、水资源保障能力进一步增强,加快石油战略储备及管道运输重点工程建设,节水城市体系基本形成。信息基础设施建设进入全国先进行列。

坚持协调发展。区域协同,城乡一体,军民融合,物质文明精神文明并重,经济社会发展与生态文明统筹,发展的协调性进一步增强。突破滨海、提升市区、开发两河协调推进,有序实现90万农业转移人口落户城镇,70万城中村、城边村居民完全市民化。完成9万套棚户区改造任务。实施农村社区服务中心建设三年行动计划,全市663个农村社区服务中心全部达到“一厅一校十室”标准。落实省农村改厕三年行动任务,完成改厕53.9万户。农村新建、改建房屋均按7度以上抗震设防。完善智慧城市体系和城市应急管理系统。建成地下综合管廊40公里。深入实施公民道德建设工程,完善公共文化服务体系,加速文化经济融合发展。争创军民融合发展示范市、军民融合产业试点示范区。

坚持绿色发展。落实国家、省主体功能区规划,按照主体功能区定位推动发展。实施绿色建筑三年行动计划,城市规划区内新建建筑全部执行绿色建筑标准。落实绿色交通、海绵城市、节水型经济建设规划。实施循环经济发展计划,建设国家循环经济示范市。开展新一轮环保行动,扎实推进“十项重点工程”。实施节能环保重大技术装备产业化工程。实行总量和强度双控管理及逐步加严的用能、用水、用地、排放标准。实施全民节能节水行动计划,全面推行能源管理,全社会重点领域单位能耗、水耗水平明显下降。

坚持开放发展。深入实施国际化四年计划,促进“引进来”与“走出去”并重、引资与引技引智并举,推动开放向结构优化、领域拓展、效益提升转变。实施出口市场多元化战略、服务贸易提升计划和进口促进战略,重点开拓服务与技术贸易、“一带一路”沿线国家市场、大宗商品进出口等。推动招商向产业、科技、文化、城建、民生、生态环保、智慧城市宽领域拓展,突出引进世界500强及行业领军企业,力争新增50家以上。加强对“走出去”的综合服务,支持优势企业更好地开展国际并购、境外基础设施投资和能源资源合作开发,建设大宗商品境外生产、营销基地。落实融入国家“一带一路”战略工作方案,选定部分战略支点城市深度开拓。深化开放体制机制创新,分行业对接国际标准、规则,规划建设一批国际社区、学校、医院,打造国际化营商环境。深入实施开发区转型升级三年行动计划,建设转型升级示范区。

坚持共享发展。强化政策兜底,引导社会投入,增进民生福祉,让全市人民一个不落地共同迈入全面小康社会。落实脱贫攻坚规划,力争两年基本完成省定贫困人口脱贫任务。实施教育优先发展战略,建设婴幼儿养育、学前教育、基础教育、职业教育、高等教育、终身教育全面发展创新区。实行更加积极的就业政策,实施高端人才创业引领计划,打造国内最优良的创新创业政策和服务环境,力争吸引10万大学生创业,带动城镇就业60万人以上,建设创业型城市。全面推行民生综合保险,居民基本养老保险、医疗保险覆盖率分别达到92%和93%。落实医疗健康产业发展规划。公立医院率先改革,大力发展民办医疗机构,推进市、县、镇 (街)、社区医疗机构协同发展。统筹保健、医疗、食品、养老、康复,建设大健康产业发展聚集区,打造健康潍坊。

各位代表,实现“十三五”宏伟目标,是全市人民的期待,也是我们共同的责任。要始终坚持以人民为中心,把人民群众的向往作为我们追求的目标,同全市人民一道,戮力同心,砥砺奋进,共同开创更加幸福美好的明天!三、2016年政府工作安排

2016年是全面建成小康社会决胜阶段的开局之年,是推进结构性改革的攻坚之年。我们要按照中央、省、市委的决策部署,认真贯彻落实宏观政策要稳、产业政策要准、微观政策要活、改革政策要实、社会政策要托底的总体思路,坚持目标导向和问题导向并重,大力推进结构性改革,落实“去产能、去库存、去杠杆、降成本、补短板”五项重点任务,推动工作指导重大转变,进一步提高发展质量和效益,努力实现“十三五”良好开局。

今年全市经济社会发展的主要目标是:地区生产总值增长8%左右,固定资产投资增长13%左右,社会消费品零售总额增长11%左右,进出口总额增长 4.5%左右,一般公共预算收入增长9%左右,城镇登记失业率控制在3.8%以内,居民消费价格上涨2.5%左右,城镇、农村居民人均可支配收入分别增长 8%和8.5%以上,全面完成省下达的年度节能减排降碳约束性目标。

(一)推进产业升级。认真落实22个产业转型升级实施方案和年度计划,把龙头企业培育、主体园区建设、产业平台搭建、对外招商合作、产业基金及政策支持等任务措施落到实处,加快构建现代产业体系。

落实品牌农业发展计划。鼓励以镇(街)、社区、村和规模经营主体为单位注册农业商标,组建经济组织,规范质量管理,开展“三品一标”认证。支持引进国际农产品质量认证机构,开展认证工作。扩大产地证明和准出、准入制度覆盖面。启动农村新型经营主体提升三年计划,促进各类合作组织更加紧密合作、经营大户科学经营、龙头企业规范化改制。支持扩大土地托管规模,开展全产业链托管服务。支持城市资本、外资下乡发展现代农业。开展化肥农药使用量零增长行动和耕地轮作休耕试点。健全农业对外深度合作机制,办好中美、中荷、中以农业合作示范项目和中韩农业认证互认试点。实施“互联网+农业”专项计划,大力发展智慧农业,搭建农业电商高端平台,建设农产品质量管理闭环系统。开展现代节水农业示范镇创建活动,年内20%的镇(街)达到节水农业标准。

做大战略性新兴产业。落实6个新兴产业发展实施方案,抓好总投资1249亿元的389个项目,新兴产业主营业务收入增长达到13%以上。电子信息产业,突出抓好光电产业园、山东测绘地理信息产业园等特色园区,培强做大歌尔声学、浪潮华光、新海软件等龙头企业,做大可穿戴设备、虚拟现实等新一代互联网终端产品及配套产业。智能装备产业,发展壮大帅克、迈赫、华创等龙头企业和3D打印技术创新中心,重点突破精密减速器、伺服电机、高精度传感器等核心技术,培强做大通用机器人、高档数控机床、工业机器人及自动化生产线等产业。生物基新材料产业,突出抓好总投资75.4亿元的产业化集群项目,今年建成投产兰典生物丁二酸及PBS产业化、3万吨纤维素纤维等重点项目,支持恒天集团建设新材料产业园。新能源及电动汽车产业,强化福田、泰汽、凯马、比德文等龙头企业带动,重点突破高速电动轿车牌照、插电式混合动力系统与自动变速器、汽车电子等关键环节和核心技术,建设好山东互联网汽车产业基地。

传统优势产业是我市产业的支撑、转型升级的基础,也是供给侧结构性改革的重点和难点。要坚持市场化机制、中高端目标、国际化视野,逐一敲开核桃,一业一企一策,扎实有序推进。改造提升一批。全面对接产业中高端和市场短缺供给,寻标对标,全面提升。组织实施好400个重点技改项目,促进产品、装备、技术、工艺、品牌换代升级。扎实开展化工行业整治,落实“四个全部”要求,推进昌邑化工原材料基地及中化弘润、鲁清石化基地等27个重点项目建设,推动重点园区优化升级。汽车及动力机械产业,完善整车、整机产品专业化协作配套链条,加快推进福田汽车及配套产业园、潍柴动力工业园扩能升级、林德液压国产化、盛瑞8AT变速器及配套产业园等49个重点园区和项目建设。纺织服装产业,加快时尚化、个性化、品牌化步伐,支持建设行业设计中心、孵化中心,加大品牌并购运营力度。食品产业,食品谷总部一期形成总部、培训、研发、孵化、展示交易、品牌运营主体功能,东亚畜牧产品交易所、中美食品中心、中荷农业创新及人才培训中心、中以现代农业研究院年内投入运营,加快30万头胴体牛进口加工分销等11个重点项目建设。优化重组一批。制定促进产业更新办法,鼓励传统企业就地转产发展新兴产业、制造企业就地发展服务业或2.5产业。支持企业开展市场化重组、并购和国际合作,支持龙头企业发展产业集团,组建产业联盟和技术创新联盟,领办、合办产业园区。有序转移淘汰一批。抢抓国际产能合作和国家去过剩产能机遇,引导低效产能向低成本地区有序转移。鼓励有条件的镇村布局特色产业园承接优势产业转移。制定实施“三高一危”企业退出计划,严格禁止新上“三高一危”项目。加快新一轮“退城进园”,年内取得重要阶段性成果。对资不抵债、扭亏无望的僵尸企业,积极稳妥地推进重组整合或退出市场,加快实现市场出清。

启动新一轮服务业跨越发展计划。推动生活性服务业向精细化和高品质转变,生产性服务业向专业化和价值链高端延伸,九个重点服务业增加值综合增长率达到 12%以上,力争服务业占比再提高2个百分点左右。优先发展总部经济和楼宇经济,潍坊总部基地一期全面招商运营,总部基地二期、食品谷总部二期基本建成。继续引进各类企业总部及分中心,支持各类公司在潍坊开展专项交易结算。支持发展孵化、电商、金融、众创、养老等特色楼宇。落实电子商务发展实施方案和人才培养年度计划,规划建设特色电商产业园,与阿里巴巴、京东等重点企业合作建设互联网潍坊产业带,重点突破农业、工业品、文化及跨境电商,打造属地化互联网结算平台,办好“首届世界互联网+农业大会”。实施物流业发展规划,统筹推进综合物流基地和海港、空港、高铁、电商四个专业物流园区建设,重点打造航空物流、食品冷链物流、同城配送、快递物流四个体系,引进培育一批高端物流骨干企业。

适应产业跨界融合、要素整合大趋势,启动实施农业产业化提升工程、制造服务化推进计划,促进农产品加工走向中高端,农业、制造业向产业链两端延伸,发展创意设计、科技信息、电商物流、金融租赁、整体解决方案等,提升产业附加值。制定实施“品牌潍坊”五年行动计划,落实城市及地域、产业(行业)、企业、产品品牌培育具体措施,打造“品牌潍坊”。制定实施重点产业(行业)“互联网+”行动计划,促进互联网与经济社会各领域深度融合。实施新一轮主辅分离工程,鼓励企业(机构)把内设服务机构分离为独立法人,在市场中发展壮大。启动产城融合发展示范工程,支持滨海区争创国家产城融合发展示范区。

(二)扎实推进新型城镇化。深入实施“三大战略”。突破滨海,突出陆海统筹、产城一体,推进大家洼、央子两大城镇组团和海洋科技大学园、中央商务区等 9大片区建设,促进海洋化工、海工制造等6大特色海洋产业转型升级,加快24个重点项目建设。提升市区,深入落实“13364”总体要求,实施中心城区规划、交通、产业、功能等“七项提升工程”,全面实施“六大服务功能”提升方案,突出抓好178个重点项目建设。每个街道选择一个社区建设“四全一特”品牌社区示范工程。开发两河,全面实施“一区三带”行动,加快推进140个重点项目建设,加强生态绿化、南部山区保护开发,突出抓好沿岸堤顶公路规划建设,推进峡山水库增容等水资源开发利用工程。

扎实推进“三个市民化”。加快户籍制度改革,全面实施居住证制度,年内实现15万外来务工人员落户城镇,实现基本公共服务城镇常住人口全覆盖。启动实施城镇棚户区改造三年行动计划,年内改造棚户区3.89万套以上,开工中心城区老旧小区改造2.5万户,实现13万城中村和城边村居民完全市民化。继续推进农业转移人口就地城镇化,将非农经济和就业超过70%的村庄和人口纳入城镇管理,年内就地转移农业人口3万人。

统筹县城、小城镇、农村社区发展。实施县域经济上档升级行动,促进县域经济上档进位。开展小城镇创新发展示范行动,落实扩权强镇措施,加快培育一批小城市。按照“一四六”标准,完善小城镇基础设施和公共服务功能,全面推行“镇区同建”。新建和改造提升农村社区服务中心166个。改造农村危房900户。完成农村2.5万户低电压治理。试点改造农厕14.9万户。

(三)扩大有效投资,加快城乡基础设施建设。当前,投资仍是补齐短板、增加有效供给、引领转型发展的关键措施。完善项目协调机制,落实项目建设主体责任,对规划的“十三五”期间总投资1.65万亿元的1685个项目逐一落实开工时限,提前做好开工准备工作,确保按期开工、顺利竣工。上半年开工412 个,年内开工701个。全面推行“裁执分离”,依法征迁,破解发展瓶颈。用足用好中央、省各项政策,完善财政资金、政府基金综合利用机制,促进商业信贷规模稳定增长,最大限度用好政策性信贷,更大力度利用社会、境外资本,大力发展直接融资,扩大全社会融资总规模。市、县两级普遍设立PPP中心。

加快城乡重大基础设施建设。加快济青高铁、潍莱高铁、潍日高速、青银高速扩容潍坊段及朐沂铁路建设,做好京沪高铁东线前期工作。加快潍坊港17个5万吨级泊位建设和机场改造、迁建、运营。启动中心城区交通提升工程,基本完成市区2条以上快速通道,开工建设东、南2条新外环路。建设中心城区重点拥挤路段地下通道(过街天桥),全面启动城市地铁前期工作。开工建设城海轻轨,完成市区至滨海快速通道主体工程,同步推进BRT建设。加快推进“特高压入潍”工程。编制完成节水城市、海绵城市建设总体规划和重点领域专项规划,健全节水协调推进机制和政府规章,开展全民节水行动,建设一批节水示范企业和示范镇 (街),力争尽快实现用水总量零增长并逐年下降。建成市云计算中心、大数据中心、云支付中心和“超级WiFi”无线城市网,搭建“物联城市”基础框架。完成市级政府重点部门平台整合。

(四)推动大众创业万众创新。全面落实市委《关于深入实施创新驱动发展战略加快建设创新型城市的意见》,成立创新创业委员会,健全工作机制,统筹推进理论创新、产业创新、技术创新、管理治理创新、发展模式创新。大力推广潍柴动力、歌尔声学、福田雷沃、豪迈科技、盛瑞传动等市内外先进经验。突出科技创新。强化高新区在促进高新技术产业化中的试验、引领、承载作用,全面优化政府产业基金支持机制,加速科技成果产业化。实施“金桥计划”,建设专利及技术成果路演中心和交易市场,发展一批技术转移中心和技术经纪组织,建立双向开放的技术转移机制。规划建设市科教创新园。加快福田汽车研究院、华大基因潍坊分院、北京大学分子育种(潍坊)试验室等平台建设。建设市工业研究院。依托重点行业龙头企业和骨干院所,统筹规划建设产业创新平台和人才团队,配套规划建设一批科技孵化器、加速器。运用市场化机制改造提升政府办创新创业孵化器,年内新增科技企业孵化器20万㎡以上。扩大科技开放合作,建设运营好重点国家、地区技术转移中心和中以跨境多币种科技创新基金,开展多形式专项对接活动。支持企业设立境外研发中心、申请国际专利、注册国际商标。实施创新金融发展专项计划,加大政府投入及创新力度,促进各类风险投资、天使基金、种子基金聚集发展、高效利用。实施“小升高”计划。高新技术产业产值占比再提高2个百分点左右。积极参与争创山东半岛国家自主创新示范区。

加快构建以众创空间、众创政策、众创服务、众创制度为重点的众创体系,发展众创、众包、众扶、众筹等创新新模式。实行“鸢都创业证”制度,吸引更多的人才创新创业。鼓励建设创新工场、车库咖啡等新型孵化器。支持市内各大中专院校创办众创空间、兴办科技型企业。

制定实施年度人才计划,把引进高层次人才与引进实用人才结合起来,继续引进一批“高精尖缺”人才,启动实施金融、设计、法律、外语、物流等实用人才千人计划。企业家是全社会最宝贵的人才资源之一。要在全社会大力弘扬企业家精神,提升企业家文化,营造支持企业家干事创业的浓厚氛围,打造一支具有国际视野、永不满足、创新创业、富有社会责任的宏大企业家队伍。

(五)深化改革、扩大开放。以供给侧结构性改革为根本,加大重点领域和关键环节改革力度。农村改革,深入推进现代农业综合改革和供销合作社综合改革试点,深化农村产权制度改革,建立市县镇一体化的农村产权交易体系。启动实施职业农民培训计划。探索建立质量效益主导型农业发展新机制。财税改革,落实“营改增”扩围改革政策。合理调整事权和支出责任,实现部门、单位预决算公开全覆盖。完善财政专项资金市场化运作机制。健全政府债务管理机制,加大债务置换力度,严防债务风险。金融改革,深化银企互利合作新机制,清理企业各种不合理负担,提升服务水平。实施资本市场开拓计划,开展新一轮“个改小、小上规、规改股、股上市”计划,年内35%以上规模企业完成改制并制定直接融资计划,新增境内外上市、挂牌企业30家以上,15%以上改制企业实现直接融资。扩大农村合作金融试点范围。加快齐鲁农村产权交易中心、基金谷、文化艺术金融试验区、金融市场等综合平台建设运营步伐。大力发展融资性担保业,加快企业互保联保贷款置换,加大商业银行不良贷款核销和处置力度,严厉打击恶意逃废银行债务行为,严控金融风险。资源要素配置改革,全面推行能源管理,探索实施用能、用水总量控制和新增规模竞争性办法,探索实行环境容量、排污权公开交易。优化公共资源招标、挂牌、采购等市场化配置机制。实施促进结构优化的价格、土地、财税政策,稳妥推进水、电、天然气等资源能源价格改革。国有企业改革,实行一企一方案,推进产权多元化和混合所有制改革,全面建立“三会一层”治理结构,有序推进竞争性领域国有股权退出。完善市级平台公司考核机制,实现市场化运作与服务大局有机统一。大力发展民营经济。落实鼓励社会资本进入社会事业领域政策,支持民营企业参与教育、医疗、文化、养老等事业单位改革和国有企业改制重组,落实民营企业在授信、评级、信贷以及土地供应等方面与国有企业同等待遇。重视新生代民营企业家培养,加大民营高级经营管理人才培训力度。改进产业引导方式。制定更为精准的产业政策,更加注重用法治思维和法治方式引导产业发展。建立更加完善的产业标准和预警机制,改进负面清单和产业目录管理,完善产业准入、管理和退出机制。加大政策落实力度和对重点产业、关键企业扶持力度。优化服务机制。完善领导干部、部门联系重点企业、重点项目制度,全面推行问题直报会商解决机制。建立政府及部门服务企业评价制度。扎实开展实体经济企业降低成本行动,研究落实降低实体经济企业成本的具体措施。

实施国际化“七项工程”,推动高水平双向开放。更大力度“走出去”。抓住“一带一路”战略机遇,确定一批战略支点城市深耕合作,支持企业开展工程承包和劳务输出。扩大境外投资,建设海外研发中心、工业园区、能源资源基地,并购国外优质资产、先进产能、技术、品牌等。支持综合保税区争创国家级跨境电商综合试验区,建设对外开放先行区。更高质量“引进来”。建设运营好东亚畜牧产品交易所、中国——东盟国际食品贸易港、进口肉类和冰鲜水产品口岸等,推动建设东亚畜牧产业合作综合平台。深化中欧城镇化伙伴关系合作。继续办好风筝会、鲁台会、文展会、中日韩产业博览会,全力争取纳入中国(山东)自由贸易试验区。落实招商工作责任制,建立精准招商新机制,推动招商工作向宽领域拓展。突出对世界500强及行业领军企业定向招商,年内力争签约引进项目10个以上。支持有条件的县市区、开发区规划建设特色国别园区。有序发展潍坊外地商会,支持在外潍坊籍乡亲为家乡发展献计出力。促进外贸稳定增长。实施“互联网+外贸”行动计划,组织开展“千企百展”活动,继续建设运营好海外潍坊商品营销中心。提升各级外贸服务平台综合服务功能,抓好进出口关键项目,推动有出口资质、无出口业务的企业突破发展,年内出口额达到134亿美元。

(六)进一步改善民生,改进社会治理。越是经济下行,越要注重保障和改善好民生,使民生更好地与经济社会发展水平相适应。今年民生支出占比再提高1个百分点,达到520亿元。认真落实全市精准扶贫三年规划,年内减少贫困人口10万人以上、省定贫困村100个以上,坚决打好脱贫攻坚第一仗。新建、改扩建 100所标准化普惠性幼儿园,新建、扩建89所中小学,力争两年内解决城镇中小学大班额问题。开展职业教育混合所有制试点,支持各类企业和境外机构兴办、参股、合办职业教育。

创建海洋科技大学,加大知名大学、国际知名教育机构引进力度。实施全民参保登记计划,稳妥推进机关事业单位养老保险制度改革。完善职工医保、居民医保和职工长期护理保险政策,稳步提高医保水平。推行民生综合保险,进一步完善社会救助体系,推动发展慈善及残疾人事业。启动建设“潍坊健康城”,打造医疗服务综合体。落实公立医院改革方案,加快建立现代医院制度和分级诊疗制度。启动基层医疗服务能力提升三年行动计划,建成规范化农村社区卫生室200所。每个县市区年内启动建设1处医养结合的新型养老综合体,新增养老床位5500张以上。推进中医进镇(街)、进社区。实施全面两孩政策。新增600处全民健身工程。

推动文化繁荣发展。加强社会主义核心价值观和“四德”教育,弘扬孝亲敬老传统美德,大力传播以王伯祥、王乐义为代表的先模精神,让潍坊优秀文化传统发扬光大。健全文明城市建设常态化机制。深入实施文化惠民工程和潍水记忆工程,做好市博物馆、美术馆、革命传统纪念馆改造建设前期工作。支持发展非国有博物馆、美术馆。启动建设中国(潍坊)创意设计产业综合集聚区,加快建设国际创意港,提升国家级广告创意产业园、软件园、十笏园文化街区、坊茨小镇、城隍庙片区等改造建设运营水平。培育引进知名文化旅游市场主体。

良好的生态环境是最基本、最重要的公共产品和民生福祉。实施新一轮环保行动年度计划的600个重点项目,全面完成年度预期目标。空气主要污染物浓度下降5%以上,“蓝天白云、繁星闪烁”天数达到240天以上。规划建设一批充电站(桩)等配套设施,购置新能源、清洁能源公交车300台以上。完成居住建筑供热计量及节能改造60万㎡以上。公共机构全面推行能源管理,单位建筑面积能耗降低2%以上。落实环境质量生态补偿政策,完善有奖举报和“一票否决”机制,对工作不力的进行约谈,对环境违法案件有一起严肃查处一起。全力争创国家森林城市。

促进社会治理创新。落实《法治潍坊建设纲要》,启动“七五”普法,建设公共法律服务体系。启动实施社会信用体系发展规划,建设市公共信用信息平台。培育发展各类社会组织。深化双拥共建,加强国防动员和国防后备力量建设。把控风险放在经济社会发展更加突出位置,持之以恒加强安全生产,全力创建国家食品安全城市,建设社会治安防控体系,提升反恐防恐能力,完善社会矛盾排查调处化解机制,健全防灾减灾体系,全面推行城市社区“四社联动”,确保全社会安全稳定。

(七)加强政府自身建设。顺应人民群众新期待,适应和引领经济发展新常态,提升引领发展、服务群众、治理社会的境界和能力。

要敢担当能担当。各级政府及其工作人员要增强大局意识、看齐意识、责任意识、表率意识,始终保持昂扬向上、奋发有为、干事创业的精神状态。带头落实创新、协调、绿色、开放、共享发展理念,以国际视野、辩证思维把握形势、谋划发展,以改革精神直面矛盾、破解难题,尊重基层和群众首创精神,虚心学习外地经验,不断开创工作新局面。要建立严格的追责机制、合理的容错机制、及时的纠错机制,支持、保护、褒奖担当者,为担当者担当!

要依法办事。落实中共中央、国务院《法治政府建设实施纲要(2015-2020)》,提升尊法学法守法用法能力和水平,自觉运用法治思维和法治方式深化改革、推动发展、化解矛盾、治理社会。严格执行市人大及其常委会制定的地方法规和决议决定,自觉接受人大依法监督和政协民主监督,广泛听取各民主党派、人民团体和社会各界意见建议。认真做好政府规章和规范性文件制定及实施工作。严格执行重大行政决策公众参与、专家论证、风险评估、合法性合理性审查、集体研究法定程序。严格规范行政执法行为,扩大基层综合执法试点范围。深化政务公开、企业和基层重大事项公开,落实经济责任审计制度,让权力在阳光下运行。

要提升服务效能。继续深化审批制度改革和商事制度改革,再依法取消一批审批项目。扩大网上申报、在线预审和远程输出批文证照比例,实现市、县、镇 (街)三级“网上联审联办”。扎实开展各种收费清理专项行动,能取消的行政收费全部取消,中介评估及收费一律实行公开竞争,行业收费逐一规范,严肃查处违规收费行为。将现有24个政务服务热线整合为“12345”1个服务热线,实行“一个号码管服务”。充分运用大数据加强和改进政务管理服务。

要加强作风建设。巩固党的群众路线教育实践活动和“三严三实”专题教育成果,坚持正风肃纪、正本清源、正道直行,把党的纪律和规矩挺在前面,持之以恒落实中央八项规定精神和省市有关规定,坚决纠正“四风”。深入开展党风廉政建设和反腐败斗争,严肃查处各种违法违纪案件!

各位代表,新时期,新阶段,新目标,新期待。让我们更加紧密地团结在以习近平同志为总书记的党中央周围,在中共潍坊市委的坚强领导下,奋发有为、锐意进取,为实现全面建成小康社会宏伟目标,加快建设富裕文明新潍坊而努力奋斗!
